# Improving the inclusion of an under-served group in trials: development and implementation of the INCLUDE Impaired Capacity to Consent Framework

**DOI:** 10.1186/s13063-024-07944-x

**Published:** 2024-01-25

**Authors:** Victoria Shepherd, Katherine Joyce, Amanda Lewis, Samantha Flynn, Madeleine Clout, Brittany Nocivelli, Jeremy Segrott, Shaun Treweek

**Affiliations:** 1 Centre for Trials Research, Cardiff University, 4th floor Neuadd Meirionnydd, Heath Park, Cardiff, CF14 4YS UK; 2 Bristol Trials Centre, Bristol Medical School, University of Bristol, Bristol, UK; 3 CEDAR (Centre for Educational Development, Appraisal and Research), University of Warwick, Warwick, UK; 4 Division of Population Medicine, School of Medicine, Cardiff University, Cardiff, UK; 5 Health Services Research Unit, University of Aberdeen, Aberdeen, UK

**Keywords:** Trial methodology, Inclusivity, Under-served groups, Adults lacking capacity to consent, Cognitive impairment

## Abstract

**Background:**

For the potential benefits of trials to reach all that they should, trials must be designed to ensure that those taking part reflect the population who will receive the intervention. However, adults with impaired capacity to consent are frequently excluded from trials — partly because researchers are unfamiliar with the legal and ethical frameworks and lack the necessary methodological expertise. Researchers identified a need for guidance on designing more inclusive trials. Building on the NIHR INCLUDE initiative, we developed the INCLUDE Impaired Capacity to Consent Framework to help researchers design inclusive trials.

**Methods:**

The framework was developed over five phases: (1) establishing the scope and content of the framework and adapting the INCLUDE Ethnicity Framework for this population; (2) scoping the relevance of the framework to different populations and piloting in a range of trials; (3) consulting people living with impairing conditions and carers to explore their views about the framework and identify missing content areas; (4) refining the framework; and (5) the development of an implementation toolkit of resources to support researchers using the framework.

**Results:**

The framework has two parts: a set of four key questions to help researchers identify who should be included in their trial, and a series of worksheets covering intervention design, recruitment and consent processes, data collection and analysis, and public involvement and dissemination. It is supported by a summary of the ethical and legal frameworks and a website of resources on capacity and consent. Implementation resources include infographics and animations, a library of completed frameworks, and facilitated workshops for researchers.

The framework and toolkit were launched at a webinar (November 2022), with polling demonstrating an increase in attendees’ awareness about research involving adults lacking capacity. A post-webinar survey found that stakeholders viewed the framework and toolkit as valuable tools to facilitate greater inclusion of this under-served population in trials. The framework is available online: https://www.capacityconsentresearch.com/include-impaired-capacity-to-consent-framework.html.

**Conclusions:**

The INCLUDE Impaired Capacity to Consent Framework and implementation toolkit can support researchers to design more inclusive trials and other types of research studies. Further engagement, including with funders who are key to ensuring uptake, and evaluation is needed.

**Supplementary Information:**

The online version contains supplementary material available at 10.1186/s13063-024-07944-x.

## Background

Randomised controlled trials (RCTs) play a vital role in evaluating the safety and effectiveness of health and care interventions — including medical treatments and services — and generating practice-changing evidence [1]. However, some populations are routinely excluded from trials, which often means little is known about which treatments are safe and work best for these under-served groups [2]. Ensuring that populations included in RCTs actually reflect the groups the intervention is intended to benefit is key to ensuring that the intervention is safe and effective in these populations, and to address the health inequalities that many of these groups experience [3]. One example of an under-served group is people who have cognitive impairment and are unable to provide consent to take part in a trial [4]. Cognitive impairment may be due to a neurodegenerative condition such as dementia, an acute illness such as a stroke, or may be experienced by people with a learning disability, a mental health condition, or those at the end of life. Trials involving adults with impaired capacity to consent encounter a range of ethical, legal, and methodological challenges, resulting in these populations frequently being excluded from research [5].

### Exclusion of adults with impaired capacity to consent

Ethical concerns about the inclusion of people considered ‘vulnerable’, including those with impaired mental capacity, have been described as a major barrier to research in areas such as palliative care [6]. Selection bias based on participants’ (in)ability to provide consent has been reported in trials in a wide range of clinical conditions including stroke [7] and aphasia [8], and in populations including older people [9, 10], with negative consequences for these excluded groups as a result. For example, as an older population, one in three patients with hip fractures have concomitant cognitive impairment [11]. This group have a substantially higher postoperative mortality risk compared to patients without cognitive impairment [12], yet systematic reviews found that 8 out of 10 RCTs evaluating the management of hip fractures [13] and of rehabilitation interventions [14] exclude or ignore this population. There is a similar picture in emergency research where despite approximately 40% of older adults presenting to emergency departments having cognitive impairment [15], this population is excluded from 25% of RCTs in emergency care [16]. Additionally, trials that are designed to include adults with impaired capacity to consent frequently struggle to obtain ethical approval and to recruit and retain participants [17, 18].

### Improving inclusion of this under-served group

Trials must be better designed so that they are more inclusive of groups that are under-served by research [19]. Improving the inclusion of under-served groups in research is a priority strategic area for international research funders including in the UK and USA [20, 21]. As part of the UK’s response to this international drive towards more inclusive research, the National Institute for Health and Care Research (NIHR) recently commissioned the INCLUDE initiative which developed guidance to help researchers to design more inclusive trials [2]. INCLUDE called for the development of tools to help researchers to design clinical studies that effectively recruit and retain such groups [2]. This led to the development of the INCLUDE Ethnicity Framework which aims to help researchers to design a trial that is inclusive of ethnic minority groups [22]. The next phase of INCLUDE is to develop tools and initiatives to improve the inclusion of other under-served groups in trials, including adults with impaired capacity to consent.

Our previous research found that researchers struggle to design and conduct trials involving people with a cognitive impairment due the complex challenges involved, describing a lack of knowledge and support to help them overcome these challenges [17]. They identified a need for more guidance on how trials can be designed to ensure that people with impaired capacity can participate in, and benefit from, research.

Working with researchers and people affected by capacity-affecting conditions and their carers, we developed the NIHR INCLUDE Impaired Capacity to Consent Framework to help researchers to design trials that are more inclusive of these groups [23]. Evidence from the development and evaluation of a previous INCLUDE framework — the Ethnicity Framework — showed that implementation activities are needed to help researchers to use a new framework in practice and are key for it to achieve similarly successful impact [24]. In this paper, we firstly outline the methods used in the development of the framework and the theoretically-informed implementation project that aims to support researchers to use the framework to design more inclusive trials in the future. We then outline the contents of the framework and implementation toolkit (see the “Results” section) alongside providing guidance and recommendations to support researchers to use them in practice. The framework and toolkit are available online: https://www.capacityconsentresearch.com/include-impaired-capacity-to-consent-framework.html

## Methods

### Development of the INCLUDE Impaired Capacity to Consent Framework

The development of the framework was led by members of the Inclusivity sub-group of the MRC-NIHR Trials Methodology Research Partnership (TMRP) Trial Conduct Working Group in the UK. It was carried out in conjunction with researchers from relevant specialties, people living with capacity-affecting conditions, and their carers. It also builds on a wider programme of research exploring the ethical and methodological issues around the inclusion of adults lacking the capacity to consent in research, and the development of interventions to address the challenges (CONSULT) [25]. As part of an implementation project, underpinned by theoretical approaches to the implementation and sustainability of interventions, we then worked with a range of stakeholders and people with lived experience to develop a toolkit to help researchers to implement the framework in their work.

**Fig. 1:**
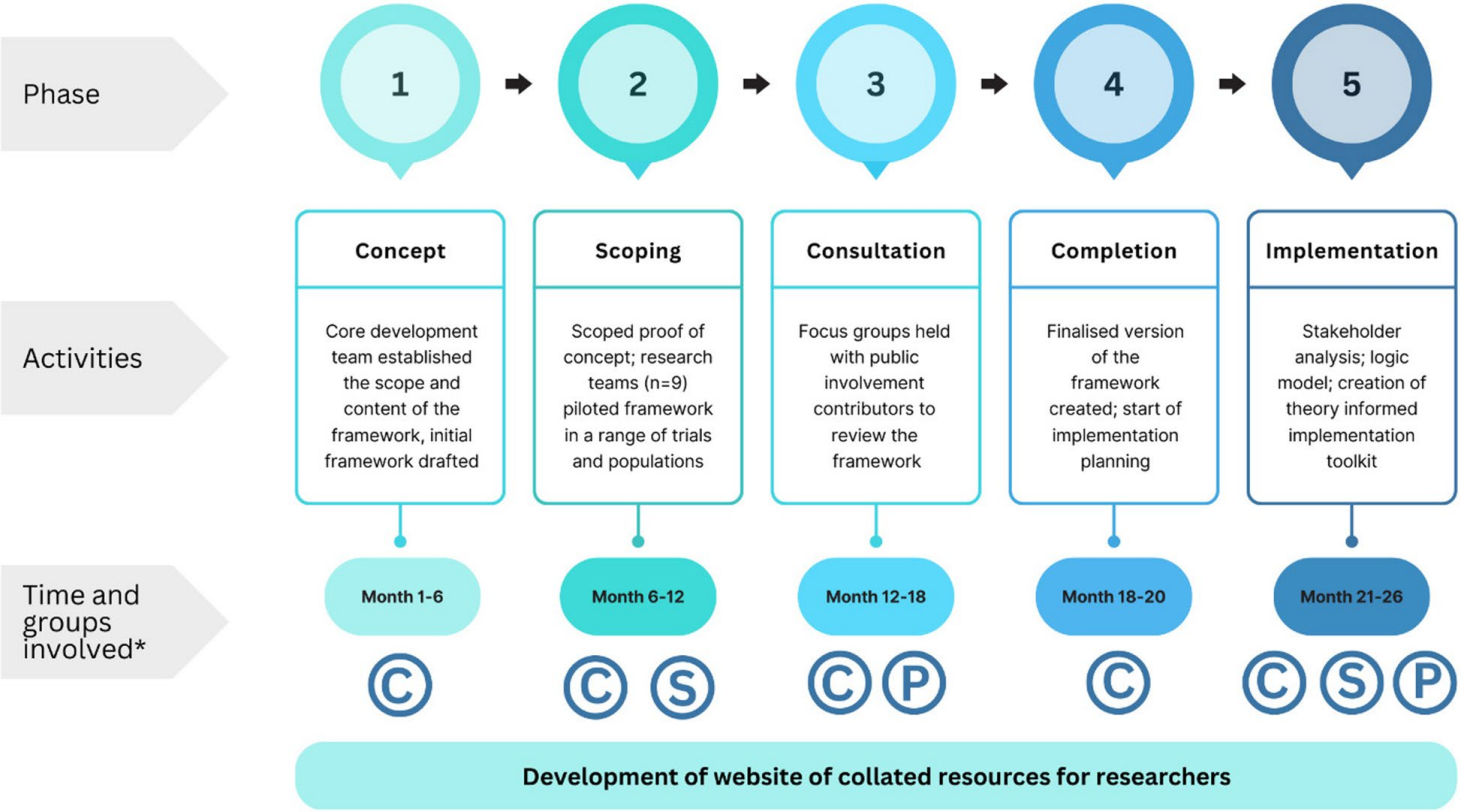
INCLUDE Impaired Capacity to Consent Framework development process. *Groups involved: C, core development team; S, stakeholders; P, public involvement contributors

The development process (see Fig. 1) comprised five phases: (1) an initial phase established the scope of the framework and adapted the INCLUDE Ethnicity Framework structure for this population; (2) a ‘proof of concept’ phase explored the relevance of the framework to different populations through stakeholder consultation and piloted the framework in a range of studies and settings; (3) a consultation phase explored the views of carers and people living with capacity-affecting conditions about the framework and identified missing content areas; (4) the framework was refined based on the feedback received, and (5) an implementation phase led to the development of a theoretically-informed toolkit of resources to support research teams to use the framework.

### Phase 1: establishing the scope and content of the framework

Development of the framework started during the first half of 2021 with a core development group (VS, KJ, SF, MC, AL) who have experience in conducting trials in a range of populations who experience impaired decision-making. The group held a series of online meetings to discuss the scope and content of framework, the feasibility and utility of adapting the INCLUDE Ethnicity Framework [22], and to iteratively develop the first draft version of the framework. The four key questions from the INCLUDE Ethnicity Framework [22] were retained, although Q4 (which asks whether the intervention/comparator will make it harder for people with impaired capacity to take part) was expanded to explicitly include retention. This was considered necessary in order to reflect circumstances where a participant’s capacity is lost during trial participation as this often results in participants being withdrawn from a trial unless it has been designed to take account of this.

The framework is intended to apply to all populations with conditions or disabilities which may impair their capacity to consent to the trial (or who may lose capacity during the trial). Capacity may be impaired as a result of the condition or disability that is the focus of the trial, or the impairing condition or disability may be co-existing with the condition that the trial is focused on. It may arise from an acute event leading to a sudden loss of capacity, from a long-term condition or disability, or be a combination of the two. Therefore, the challenges needing to be addressed will vary with each trial, and the communication and capacity support needs of each population (and between members of each population) will differ.

The heterogenous nature of individuals and groups who may experience impaired capacity to consent, and therefore the contextual issues that trials involving these populations encounter, was forefront in these discussions. It was also acknowledged that trials may involve a broad range of conditions, intervention types, and settings. Discussions focused on whether a single framework could take account of these contextual differences, or whether context-specific versions were needed. However, the commonality of many of the underlying principles and challenges [17] led to the decision to develop a single framework. It was agreed that in the next phase of the project, we would explore whether the framework was applicable to a range of contexts and to consider whether additional context-specific implementation tools or resources might be needed.

In order to explore contextual factors affecting the applicability and utility of the framework in different populations, we approached national research leaders from the UK (e.g. NIHR CRN National Specialty Leads, Health and Care Research Wales (HCRW) Specialty Leads) who work with populations where there may be challenges around consent. Contact details were obtained through funders’ websites that listed relevant specialty leads. Those kindly agreeing to provide feedback were emailed a copy of the draft framework, an appendix outlining a summary of the legal frameworks governing research with adults who lack capacity, and a background document about using the framework. Specialty leads were asked to provide feedback either via an online form or by email and to focus on a series of four questions (see Table 1). They were also able to add any specific comments about the contents directly into the documents and return them by email.

**Table 1:** Questions used to explore the initial scope and content of the framework

1) What are your views about the usefulness of a framework for designing trials to include adults with impaired capacity? Are there any types of trials or particular populations or settings you think it would be more, or less, useful for?	
2) What are your views about the current format (4 key questions, worksheets for Q2–3 and a worksheet to identify measures needed)?	
3) What are your views about the contents? Is anything included that shouldn't be, is anything missing that should be added?	
4) What are your views about the supporting information (background document, appendix on legal frameworks, links to resources)? Is anything included that should not be, is anything missing that should be added?	

Feedback was obtained from research leaders (*n*=5) across a range of specialties including emergency and critical care, stroke, and ageing. The framework and contents were generally viewed as an important and valuable tool, with the legal summary being viewed as particularly useful. Although the rationale for using the four key questions with corresponding worksheets was not clear to one. Suggestions were provided about how to take account of different populations and settings in order to maximise the utility of the framework, including emphasising that inclusion of all groups is the starting point. Other suggestions included providing completed examples of the worksheets. One questioned whether the worksheets could be tailored further (perhaps by the user) to the study setting to help focus on the key factors that will impact on inclusion need to be addressed.

The core development group discussed the feedback and considered that the utility of the framework had been established in principle. The feedback was used to refine the contents of the framework ahead of Phase 2.

### Phase 2: ‘proof of concept’ scoping and piloting the framework

‘Proof of concept’ [26] was defined in this project as a process to establish the utility of using the structure of the INCLUDE Ethnicity Framework to design trials involving this population, and whether it was feasible to apply a single framework to a range of studies involving different populations. Invitations to pilot the framework were distributed in Autumn 2021 through UK research networks (e.g. UKTMN, South West Research Hub, learning disability researcher groups), existing collaborators (e.g. Trial Forge https://www.trialforge.org/), and via social media (Twitter/X). Researchers who were interested in piloting the framework were asked to email the lead author to express their interest.

To guide researchers or research teams who agreed to pilot the framework, we developed a set of standard instructions. These asked teams to select a trial which may include adults with impaired capacity to consent, which could be a real (ongoing or completed) trial, a trial that was currently being developed, or a hypothetical trial. Researchers were asked to go through the framework and apply it to their trial and complete and return the worksheets. They were asked to comment on any wording or sections that they found unclear or which required amending, and whether they found any areas particularly helpful or unhelpful.

A total of 20 expressions of interest (EOI) were received by email, who were subsequently sent further information. The framework and instructions were sent out to 13 researchers/research teams who subsequently agreed to pilot the framework. Of these, 11 provided comments or feedback. Brief characteristics of the studies or research contexts which informed the basis for their feedback are shown in Table 2.

**Table 2:** Characteristics of studies/contexts used to pilot the framework

Study characteristic	No. studies/contexts
**Population/condition**	
Dementia	1
Learning disabilities	1
Genetic disorders	1
Older adults in community	1
Trauma	2
Critical care	1
Aphasia	1
Stroke	1
Combination of populations	2
**Trial type**	
Interventional/non-CTIMP	5
CTIMP	1
Surgical	2
Non-trial (genotyping data only)	1
Not specified/generic	2

Researchers could pilot the framework and provide feedback either as an individual or as a group. In addition to providing information about the trial used to pilot the framework (e.g. whether hypothetical or real, a brief summary of the trial), researchers were asked to provide responses to a series of questions (see Table 3) which were based on those used in Phase 1 and to return the completed framework document. Of the 11 who provided feedback, five returned worked examples of the framework, one of which was only partially completed. The questions and responses are summarised in Table 3 below.

**Table 3:** Summary of responses to questions about piloting the framework

**What are your views about the current format (four key questions, worksheets, appendix)?**	
**• Length** — researchers were supportive of the use of the questions and worksheets. However, the document was considered quite long, and they felt it was important that any duplication and redundancy was removed (e.g. being able to indicate ‘not applicable’ in worksheet sections) to ensure the framework is explicit and directive. Changes to formatting could reduce some overlap and length of the document. **• Layout** — the layout was generally considered to be clear. However, some suggested changes to the flow or order of the document, such as having a header on each page or having each worksheet directly after the corresponding question (rather than having all four questions first. It was also thought useful to have an area to note down any actions or considerations alongside each section rather than only at the end of the framework, these could then be collated together.	
**What are your views about the contents? Is anything included that shouldn't be, is anything missing that should be added?**	
**• Instructions for use** — there was some confusion about the reference to the INCLUDE Ethnicity Framework and therefore which groups the framework was intended to focus on. It was suggested that general guidance be included about how long the framework may take to complete, how to best utilise it, who should be involved, and when it should be used. **• Content** — greater signposting to resources and examples were thought to be useful through the worksheets rather than just at the end. It was also suggested that more could be asked about retention/completion of follow-up, particularly in relation to long term follow-up. Earlier signposting to the appendix containing the legal summary would be particularly helpful.	
**Are there any types of trials or particular populations or settings you think it would be more, or less, useful for?**	
**• Populations** — as anticipated, there were mixed views about how the framework might apply to different populations. Researchers who were developing studies involving people with a variety of diagnoses and co-morbidities or where the majority of participants would lack the capacity to consent encountered difficulties answering some of the questions (e.g. those asking about ‘the’ population or how the severity or prevalence may differ between groups. Others who were developing studies involving older people found the framework useful even though there was a range of capacity-affecting conditions to consider. **• Context surrounding loss of capacity** — it was suggested that having two versions of the framework could be considered, such as one for studies with people with acute loss of capacity (e.g. trauma, cardiac arrest) and those whose loss of capacity is longer-term (e.g. dementia, learning disabilities). In part, this was because whilst the underlying legislation is the same, the beliefs and experiences of the people involved (and their family’s involvement) are likely to be very different. **• Study types** — There were questions about whether the framework was applicable to all types of studies. It was considered particularly useful for interventional trials rather than those not involving the recruitment of individual participants, although it (or some elements of it) may be useful across a wider range of studies including observational studies. One team wondered whether there could be different worksheets for different study designs, or a filter question, or whether it could be clearer that some questions may not be applicable to all study designs. **• Timing** — it was viewed as a very useful tool during the early stages of design and grant application. Plus, when developing finer details. One team reported that whilst many of the aspects included in the framework had already been discussed at the grant application stage, when developing the protocol, it had proved useful to help consider additional aspects that hadn’t been considered at the earlier stage. **• Ongoing use** — one team thought that it would be helpful to have as a live document throughout the course of the study, from grant application, protocol development, ethics through to recruitment and beyond. Others suggested that the worksheets relating to question 4 would act as a reminder/checklist when developing trial processes and documentation, in particular the protocol, or could act as a record for decisions made about inclusion and so be a useful accountability mechanism to monitor in trial management groups and so help keep the inclusion of under-served groups near the top of the agenda.	
**Any other comments**	
**• Positive framing** — it was suggested that centring the ethical dimensions of fairness and justice and a stronger emphasis on inclusion and ensuring participation (rather than exclusion) would be useful. The framework could potentially include a statement about why this population needs to be considered with some examples of cases where it would be unethical not to include them. **• Decision-specific nature of capacity** — teams reported that having a reminder about the time- and decision-specific nature of capacity was considered very helpful to foreground answering the questions. It was suggested that this could be more prominent or upfront in the framework. **• Time and workload involved** — one team reported that completing the framework had taken longer than the 2-h group meeting they had planned. They suggested that completing particular sub-sections with smaller groups or people with particular roles might be helpful, and then collating the sections. Another noted that it may be too big a task for junior research team members to complete alone and that training and support might be needed from the lead investigator. **• Need for additional resources** — there was a suggestion that, as it relies on some prior understanding about research involving adults lacking capacity, some researchers may be so unfamiliar with the context they may be unable to work through barriers to participation. An additional resource such as a slide set or a video could support engagement with the framework. It was also suggested that worked examples would be helpful.	

Researchers reported that piloting the framework led to a change in some of the research teams’ knowledge and attitudes towards the inclusion of people with impaired capacity. One described it as ‘an enjoyable process’ that had made them think more deeply about the issues around inclusion of adults with impaired capacity to consent and encouraged them to think of potential practical solutions that might help such as discussions with their public involvement group. Others reported that they found the guidance especially helpful as they usually excluded people who cannot consent to research (or do not have a study partner) from their studies without really considering whether they could take part. One research team reported that during the design phase of the study, they would not usually consider the nature of impaired capacity that may affect their participant population and so they found the question relating to that issue very useful as it had initiated helpful conversations.

### Phase 3: stakeholder consultation

For this project, in line with the MRC guidance for developing and evaluating complex interventions, stakeholders were considered to be individuals or groups whose personal or professional interests are affected by the framework, with patients and members of the public being considered key stakeholders [27]. Stakeholder consultation was embedded throughout the five phases of the development and implementation of the framework. Phase 3 particularly focused on stakeholders with lived experience of living with a condition that may affect memory or understanding and family members caring for someone living with a capacity-affecting condition and was conducted iteratively throughout the development of the framework and toolkit. This built on the public and patient involvement activities as part of developing the INCLUDE Ethnicity Framework [22] which was subsequently adapted to form the basis of this framework.

#### Public stakeholder consultation during development of the framework

Two discussion groups were held online in May 2022. Invitations to join the discussion groups were shared with a lay advisory group that supports a parallel project exploring adults who lack the capacity to consent (CONSULT [25]) and also disseminated by Health and Care Research Wales to their involvement community who could submit an expression of interest to the framework development team. Prior to the discussion groups, information was circulated to the four attendees which included information about clinical trials (a link to the NIHR website and an easy read guide produced by NIHR [28]) and a discussion document about ensuring trials are inclusive of people with impaired capacity to consent that was created by the team alongside an easy read version of the discussion document.

Members of the framework development team gave a brief presentation about the background to the framework, its purpose, and the contents of the draft framework. Attendees were asked for their views about the contents, what information was currently missing which should be included, and what could support families and other groups to use the framework as public involvement contributors. Discussions with the groups highlighted the importance of ensuring that information is useful to carers (e.g. avoiding terminology which may not be widely understood) and provided in an accessible format (e.g. using mixed media such as video), and ensuring diversity and inclusivity is reflected in any visual imagery used. It was also suggested that facilitating workshops with research teams and public involvement contributors would be helpful and could be a way of ‘humanising’ the issues. Attendees also had the opportunity to provide written comments on the framework document by email following the group meetings. Two members of the discussion groups provided detailed feedback.

#### Public stakeholder consultation during the development of the implementation toolkit

Following the development of draft materials for the implementation toolkit (see Phase 5), we held further consultation to seek feedback on the content and format. Online discussion groups were held in September 2022 with seven members of the public who were living with a condition that may affect memory or understanding or had experience as a carer. The groups included the four attendees from the previous discussion group together with three additional members who were identified through the same routes to ensure more diverse perspectives were represented. Draft versions of a two-page infographic and an animated explainer video were shared with the group.

Attendees discussed the presentation of the information and the use of shape and colour to illustrate concepts of ‘under-served groups’, ‘exclusion’, and ‘widening participation’ rather than photographic images. This approach had been chosen by the development team due to the challenges of accurately representing the many heterogenous populations and settings where impaired capacity to consent may be encountered. There were mixed views about the use of abstract shapes, with some suggesting it was difficult to follow, although others viewed it more positively particularly as they became more familiar with the images being used.

Feedback was provided on the optimal ordering of information. They suggested dissemination routes for sharing information about the framework and implementation toolkit and highlighted the need for a coordinated strategy or campaign for engagement with stakeholders including funders. The group also suggested it would be helpful to create additional context- or population-specific materials, such as videos with different populations sharing their diverse experiences (e.g. someone from a minority ethnic group who is living with dementia or an acute brain injury).

#### Co-production of an easy read guide to the framework

As public involvement is a key part of designing inclusive trials, an important component of the implementation toolkit is accessible information about the framework that could be provided to public involvement contributors. We worked with Thinklusive [29], a specialist designer of inclusive communications, to develop an easy read user guide for people with communication and cognitive disabilities. Creating accessible information can support people with additional support needs to understand the information being provided, including people with a learning disability, people with other types of disability and people who are not fluent in English. Using the framework document as the basis, we worked collaboratively with Thinklusive to establish the aims of the accessible version and to develop a broad outline of the content. The Thinklusive team then co-produced the user guide content and layout with the Thinklusive Advisory Group, a group of experts by experience, over several workshops.

The easy read guide consists of four sections which help to support understanding by introducing and structuring the concepts involved including an introduction to the framework and the need to design inclusive trials, a guide to clinical trials, capacity and consent to research, and the structure and content of the framework. The user guide contains links to more information and is illustrated by ‘real world’ examples of decision-making suggested by the advisory group drawn from their own experiences.

### Phase 4: refining and finalising the framework

Following the consultation, the content and format of the framework was finalised by the development team. The contents of the framework are described in the Results section below.

### Phase 5: implementation project

An implementation project was planned to identify and address any barriers to the uptake of the framework, some of which were identified during the pilot stage of the framework development and those identified by Trial Forge [22, 24] during the implementation and evaluation if its ‘sister’ framework — the INCLUDE Ethnicity Framework. As a complex intervention that is intended to disrupt current systems for designing trials [30], we recognised the need for theorising how it would work in practice, which takes account of how it interacts with the context in which it is implemented [27]. The project was underpinned by implementation science (IS) which helps to identify the contextual barriers and facilitators that enhance innovation uptake [31] and using Normalisation Process Theory (NPT) which addresses factors which affect the integration of interventions into routine work [32].

This phase of the project was led by the implementation team ((VS, ST, BN, JS) and consisted of a series of activities to identify and address the barriers to implementation of the framework. The key activities are outlined in the following section.

#### Stakeholder analysis and development of an impact plan

A stakeholder and public analysis was conducted to identify the organisations and groups who are the beneficiaries of this research, prioritise the stakeholders, and develop strategies to engage with them effectively in order to generate impact [33]. An impact plan was then developed to map these stakeholders against the planned activities that were intended to engage them in the project, to identify any barriers to engagement, and to develop tailored approaches where necessary [34]. Stakeholders included: funders of health and care research (e.g. NIHR), policy and research governance organisations (e.g. Health Research Authority), researchers, and patients and members of the public. Activities to support engagement included a stakeholder consultation (see section below).

#### Development of a logic model

**Fig. 2:**
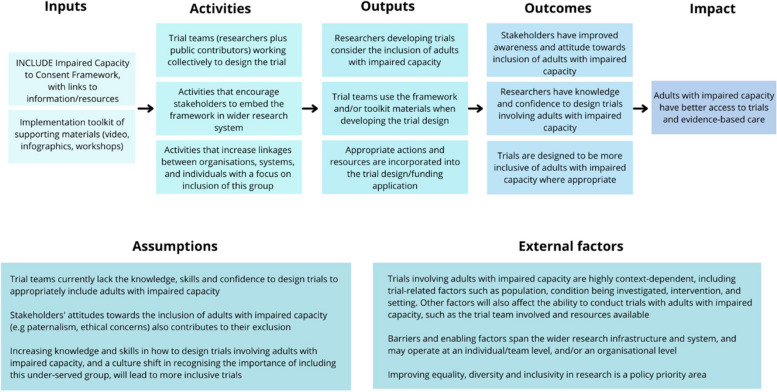
INCLUDE Impaired Capacity to Consent Framework logic model

Using theories of change, a logic model was developed to help identify the inputs, processes, outputs, and outcomes required for the successful implementation of the framework and the causal mechanisms [35] (Fig. 2). The underlying assumptions were also included in the logic model, alongside the external and contextual factors that may affect implementation. The logic model was used to identify barriers to implementation, including the need to raise awareness with researchers and patient and public involvement groups about the issues around the lack of inclusion of this population and the purpose of the framework.

#### NPT analysis for implementing the framework

The process of intervention development and implementation requires a strong theoretical foundation, with NPT being previously found to support the implementation and sustainability of interventions [32]. As a behavioural approach, NPT can help to explain how complex interventions work, identify factors that promote and prevent their incorporation into everyday practice, and ultimately lead to the point where an intervention becomes so embedded into routine practice that it is normalized [36]. NPT is considered to have four generative mechanisms: coherence, cognitive participation, collective action, and reflexive monitoring [36]. These mechanistic components are considered to have dynamic relationships between themselves and with the wider context of the intervention [32]. Using an analytical approach that has previously supported the wide-scale implementation of complex interventions [32], we conducted an NPT analysis for implementing the framework (see Table 4).

**Table 4:** NPT analysis for implementing INCLUDE Impaired Capacity to Consent Framework

NPT components	Questions to consider within the NPT framework	INCLUDE Impaired Capacity to Consent Framework
**Coherence** (i.e. meaning and sense making by participants)	Is the intervention easy to describe?	The intervention is easy to describe.
	Is it clearly distinct from other interventions?	The intervention is distinguishable from other tools such as the INCLUDE Ethnicity Framework.
	Does it have a clear purpose for all relevant participants?	It has a clear purpose, although the purpose may be unclear to some, for example it could be confused with a tool to help assess capacity to consent. To be strengthened through additional messaging such as via an explainer video and infographics.
	Do participants have a shared sense of its purpose?	The development of the framework originated in the MRC-NIHR TMRP Trial Conduct Working Group which, together with engagement with specialty leads and researchers, has provided a shared sense of purpose. To be strengthened through stakeholder consultation.
	What benefits will the intervention bring and to whom?	The framework is expected to improve the ability of researchers to design trials to include adults with impaired capacity. It will also benefit funders, research ethics committees (RECs) and other organisations through providing assurance about the quality/appropriateness of the trial design. Longer term, it will benefit people with impaired capacity through improving access to trials and generating evidence to underpin their care.
	Are these benefits likely to be valued by potential participants?	During public involvement activities and stakeholder consultations, researchers and patients/carers have stated that they value the potential benefits of the framework. Funders, RECs and other organisations have valued similar tools such as the INCLUDE Ethnicity Framework.
	Will it fit with the overall goals and activity of the organisation?	Developing research that is more inclusive of under-served populations is a high priority for both policy (e.g. Department of Health and Social Care) and organisations (e.g. National Institute of Health and Care Research, Health Research Authority). The framework supports these strategic priorities to improve equality, diversity and inclusivity in research.
**Cognitive participation** (i.e. commitment and engagement by participants)	Are target user groups likely to think the intervention is a good idea?	Based on previous experience with the INCLUDE Ethnicity Framework, researchers and other stakeholders are likely to think the framework is a good idea.
	Will they see the point easily?	Researchers and other stakeholders will see the point of the framework, but this may depend on the level of their own knowledge, ability and confidence. To be strengthened through additional messaging such as via an explainer video and infographics.
	Will they be prepared to invest time, energy and work in it?	Time-constraints and the framework length and format may impact on researchers’ willingness to invest time and energy in it. Additional messaging (e.g. via an explainer video and infographics), maximising the usability of the framework (e.g. refining the format and content) and facilitating its use (e.g. through the development of workshop materials) will be key.
**Collective action** (i.e. the work participants do to make the intervention function)	How will the intervention affect the work of user groups?	The framework will increase time and work at the initial trial design phase, but that may be recouped when the framework is revisited to inform the protocol design and REC submission stages.
	Will it promote or impede their work?	The additional time required to fully consider issues during the trial design stage may be viewed as impeding the process of developing funding application, but it may enhance the quality (and therefore its competitiveness) of the application. If the trial is funded, it may facilitate later stages, e.g. protocol design and application for ethical approval.
	What effect will it have on processes?	The framework will encourage conversation, consideration, and collaboration during trial design decisions. The actions and resources needed to improve inclusion may appear to make the trial more complex and time and resource-intensive (i.e. less ‘efficient’) as economic benefits are typically easier to measure than social/wider health benefits. However, these should be viewed as ‘missing’ costs and challenge ideas around current efficiencies as ‘doing the wrong things righter’ which needs to change. To be strengthened through additional messaging, e.g. via an explainer video and infographics.
	Will staff require extensive training before they can use it?	Most researchers will not need extensive skills and knowledge to use the framework, but they will require support to maximise their use of the framework and signposting to information and resources in order to ensure appropriate actions and resources are planned.
	How compatible is it with existing work practices?	Establishing an additional (optional) step into the process of developing a trial for a funding application is compatible with existing work practices. However, it may increase the burden on researchers at an already busy time. The impact of this will need to be monitored. This could be explored as part of a future evaluation.
	What impact will it have on division of labour, resources, power, and responsibility between different professional groups?	The framework is intended to be used collaboratively across the co-applicants, wider research team, and public contributors. It is not intended to be the work of one person. However, it may disproportionately increase the burden on some team members. The impact of this will need to be monitored. This could be explored as part of a future evaluation.
**Reflexive monitoring** (i.e. participants reflect on or appraise the intervention)	How are users likely to perceive the intervention once it has been in use for a while?	Use of this framework (and other similar frameworks) may be perceived as an important and necessary step when developing a trial, or an additional burden/hurdle to overcome as a ‘tick-box’ exercise. This will need to be monitored and could be explored as part of a future evaluation.
	Will it be clear what effects the intervention has had?	The aim is for adults with impaired capacity to have better access to trials. The effect will need to be monitored, such as assessing the number/proportion of trials appropriately designed to include this group. This could be explored as part of a future evaluation.
	Can users/staff contribute feedback about the intervention once it is in use?	Individuals and organisations will be encouraged to have ‘ownership’ of the framework through stakeholder consultations and subsequent engagement. Feedback will be sought following the facilitated workshops and via informal contact with researchers and organisations.
	Can the intervention be adapted/improved on the basis of experience?	Further work is needed to address issues around intersecting factors, and the relationship between the frameworks for different under-served groups.

Adapted from: Murray, E., Treweek, S., Pope, C. et al. Normalisation process theory: a framework for developing, evaluating and implementing complex interventions. BMC Med 8, 63 (2010). 10.1186/1741-7015-8-63

The term ‘participants’ is used in this context to refer to the target audience for the framework, including research teams who will be the primary end-users

During this analysis, a series of questions enabled us to explore how the four components of NPT might affect the uptake and use of the framework, and identify the actions needed to support its implementation. Questions such as ‘Does it have a clear purpose for all relevant participants?’ and ‘Will they see the point easily?’ led us to identify a need for an implementation toolkit to accompany the framework which would provide additional information to stakeholders about the purpose of the framework and practical support to help research teams to use it. Uncertainties about the format and contents of the implementation toolkit were then the focus of the stakeholder survey which followed this analysis.

#### Stakeholder consultation survey

Informed by the NPT analysis, we conducted a stakeholder consultation to help finalise our implementation plans. A short survey was created using Microsoft Forms (https://support.microsoft.com/en-us/forms) and shared with relevant organisations (e.g. NIHR Emergency Care Incubator, TCWG, BSG Care Home Special Interest Group, RECs) and individuals (e.g. NIHR/HCRW Specialty Leads) and via social media (Twitter/X). The survey was open June-August 2022 and consisted of a combination of multiple-choice options and open-text responses. Characteristics of stakeholders who responded to the survey (*n*=25) and their main area of interest are shown in Table 5. The survey questions are shown in Table 6 together with a thematical summary of the responses.

**Table 5:** Characteristics of stakeholder consultation survey participants

Area of interest	Academic researcher ***n*** (%)	Clinician ***n*** (%)	Clinical researcher ***n*** (%)	PPI group member ***n*** (%)	REC member ***n*** (%)
Dementia/ care home/older adults	10 (40)	1 (4)			
Emergency medicine	1 (4)		2 (8)		
Intellectual disability	1 (4)				
Intensive/critical care	2 (8)				
Primary care	1 (4)				
Clinical psychology	1 (4)				
Trauma				2 (8)	
Not specified	2 (12)				1 (4)
**Total** ***n*=25**	**19 (76)**	**1 (4)**	**2 (8)**	**2 (8)**	**1 (4)**

**Table 6:** Summary of responses to stakeholder consultation survey

**What are your views about using the framework (e.g. its value and whether you would feel capable/confident to use it)?**	
**•** Views about using the framework were provided by 96% (*n*=24) of respondents **• Positive views**^**a**^ — *n*=13 (52%) expressed only positive comments about the framework, including generic comments (e.g. describing it as excellent) and more specific comments about the framework’s clarity, useability, and usefulness **• Need for more information** — other responses included questions about how it applied to particular trial populations (e.g. critically ill patients), types of research (e.g. only clinical trials of interventions) and specific issues (e.g. surrogate decision-makers) **• Other responses** — included one comment that the framework was difficult to understand	
**What would be the potential barriers to you using the framework?**	
**•** A number of potential barriers to using the framework were reported by respondents (96%, *n*=24). Only one reported there being no potential barriers to using the framework **• Lack of understanding about the framework** — this was reported as a general barrier by some respondents (20%, *n*=5), including not understanding the target audience, the purpose of the framework, or its use and application, or a lack of understanding and knowledge about who is to be considered as having impaired capacity **• Lack of time** — this was a common potential barrier to using the framework, reported by 6 (24%) of respondents, was the time required to work through it. One respondent suggested giving an indication of how long each worksheet might take to complete. Another response included the potential difficulty in “persuading others in study team that it is something worth putting time and effort into” **• Lack of support from influential stakeholders** — this was also cited as a potential barrier by respondents (12%, *n*=3). For example, whether the framework is considered, or accepted, by funders, Research Ethics Committees (RECs), or members of the public **• Other potential barriers to using the framework** — these included whether it would help identify practical solutions to the issues raised, ease of access to the worksheets, or a lack of support and guidance to use the framework	
**Which of these implementation toolkit items would be likely to help you to use the framework?**	
**• Implementation toolkit items in order of importance**^**b**^**:** - Worked examples of the framework (*n*=22, 88%) - Links to resources to help with any actions identified (*n*=19, 76%) - Infographic with key messages (*n*=17, 68%) - Accessible information with key messages (*n*=14, 56%) - Short explainer video (*n*=12, 48%) - Interactive workshop materials (*n*=10, 40%) - Other (*n*=6, 24%), e.g. having a range of tools available **• Materials for different stakeholder groups** — suggestions were made about other ways respondents felt would help them use the framework such as producing materials for funding bodies, research design service teams, and research ethics committees **• Signposting by funders** — it was suggested that funders should either signpost applicants to the framework or embed it in their guidance for applicants **• Access to expertise** — another suggestion was that having someone in their organisation being an expert user of the framework and toolkit	

^a^Responses were considered to be positive if they contained words or phrases that were complimentary or expressed approval

^b^Participants could select more than one option from the list

Those responding to the survey could also provide the details of organisations they thought would be interested in hearing about the framework or receiving details of a webinar being planned, and responses included a range of research networks, funders, and advocacy/support groups for relevant populations. They could also indicate if they were happy to be contacted about the next stage of the framework implementation process.

### Development of an implementation toolkit to support the use of the framework

A multi-media toolkit was developed to support implementation. This included a short animated ‘explainer video’ which we commissioned an external design company to create to raise awareness with researchers and other stakeholders about the issues surrounding the exclusion of this under-served group and the purpose of the framework. This was used to develop a set of infographics to reinforce the message in increasing levels of detail and a ‘user guide’ which provides more detailed instructions about how and when the frameworks should be used (Supplementary file 1). The video, layered infographics, and user guide were intended for use on relevant websites (e.g. universities involved in the development and other relevant research networks) and to be shared via social media. Welsh language versions were also developed, and an easy read version of the user guide was co-produced (see Phase 3 for more details about the co-production process and who was involved). Collaboration with graphic design teams ensured that the toolkit materials are visually appealing and provide a professional and cohesive ‘brand identity’ to the framework and accompanying resources. The framework is supported by a website of collated resources on capacity and consent in research that has been developed in a parallel project (CONSULT [25]). The framework and implementation toolkit are available via the resources website (https://www.capacityconsentresearch.com/include-impaired-capacity-to-consent-framework.html).

### Online webinar to introduce the framework

The framework and toolkit were launched at an online workshop (November 2022) with 250 attendees comprising researchers, ethics committee members, healthcare professionals, and members of the public including public involvement contributors. The aim of the webinar was twofold - firstly to introduce the framework and explain how, when, and by whom it should be used, and secondly to highlight it as one of the methodological tools being developed that are supporting the wider strategic work around equality, diversity and inclusivity by funders (e.g. NIHR) and policy-makers (e.g. Department of Health and Social Care) both in the UK and beyond. This was achieved through a series of presentations followed by a panel discussion. A recording of the webinar is available (https://www.youtube.com/watch?v=bJt84ZjqMjc).

Of those who responded to a poll of attendees (*n*=180), responses showed that the audience consisted primarily of researchers (64%), health and social care professionals (16%), members of the public (5%), research ethics committee members (2%), and others (13%). Pre- and post-webinar polls showed an overall increase in stakeholders’ awareness about research involving adults lacking capacity, rising from a pre-webinar mean score 5.27 (‘Out of 10 how aware did you feel about the topic before the webinar?’) to post-webinar mean score 7.25 (After watching the presentations and discussion, out of 10 how aware do you feel now in applying this in your practice?’).

Following the launch, a feedback survey found that the framework was welcomed by researchers, who considered the framework and accompanying resources to be valuable tools that help facilitate greater inclusion of under-served populations when designing future trials. Questions that were asked during the webinar and in the feedback survey were collated thematically and used to create a ‘frequently asked questions’ (FAQs) document that formed an additional part of the implementation toolkit (Supplementary file 2). This included questions about how the framework applies to research conducted in different jurisdictions where the legal frameworks will differ, whether it can be used for different types of research beyond clinical trials, and how the burden of using the framework can be reduced for busy research teams.

### Workshop activities to support researchers to implement the framework effectively

As our previous research showed that research teams often lack the knowledge and methodological expertise to design and conduct studies involving this population [17, 37], we also developed more active forms of support. The development team considered that the questions included in the framework could act as a sensitising device to enable research teams to consider the barriers and facilitators, rather than solely having the framework document as the focal point. Facilitated workshops were thought to be the best way to support research teams to work through the framework questions, identify the barriers, and signpost them to tools and resources to address them. They would also provide a mechanism for the development team to observe researchers implementing the framework in practice, and to gather feedback. Therefore, as an additional part of the implementation toolkit, we developed workshop materials to support research teams’ discussions.

Over a 3-month period we piloted facilitated workshops with trial teams (*n*=4) who were designing trials involving adults with impaired capacity to consent, including those who lack capacity. Workshops were held online, lasted 1–1.5 h, and explored the frameworks questions in a range of trial contexts. Trials included those in settings where the population would predominantly lack the capacity to consent (e.g. critical care, stroke) and where cognitive impairment is prevalent in the population but the focus of the trial is a health condition unrelated to the impairing condition (e.g. care home residents living with diabetes, people living with cancer and multiple long term conditions). They also included different trial designs (e.g. RCTs, platform trials), types of interventions (e.g. medicines, medical devices, complex interventions), and at different stages of development (e.g. funding application, protocol development). Each workshop was led by two members of the framework project team (VS, BN) and involved a range of research team members including chief investigators, trial managers, research nurses, and public involvement contributors. Some teams were experienced in conducting research involving adults with impaired capacity and for others it was their first study involving this population. Informal feedback was collected from research teams and the framework project team created summaries of the discussions following each workshop. The workshop materials were iteratively refined where needed.

The workshops received overwhelmingly positive feedback. Research teams described it as being a useful and informative experience, which provided them with a lot to think about — including those who were experienced in research involving adults with impaired capacity. They reported that the discussions and information arising from the workshops had enabled them to incorporate the additional actions and resources identified as being necessary to conduct an inclusive trial into the funding application or include the actions and processes in the protocol being developed. Our observations highlighted the contextualised nature of the issues arising when designing trials to include adults with impaired capacity, and therefore which aspects of the framework may be more or less relevant for each trial. For example, Q3 of the framework which relates to the intervention/comparator and how people with impaired capacity may respond to or engage with it may be less relevant in critically ill patients who are sedated and ventilated compared to a complex intervention involving care home residents. By contrast, other items such as worksheet C which explores issues around consent and the involvement of consultees and legal representatives are relevant to all trials although the exact consent process and timings involved may well vary between different trial contexts. The need for further education and training on the legal and practical issues surrounding capacity and consent in research was commonly highlighted by research teams.

## Results

The INCLUDE Impaired Capacity to Consent Framework has been developed for researchers designing trials involving a range of populations where the capacity to consent may be impaired. A multi-media implementation toolkit (https://www.capacityconsentresearch.com/include-impaired-capacity-to-consent-framework.html) has been developed to raise awareness about the framework, support researchers to use it, and engage with stakeholders including funders who will be key to ensuring uptake of the framework.

**Fig. 3:**
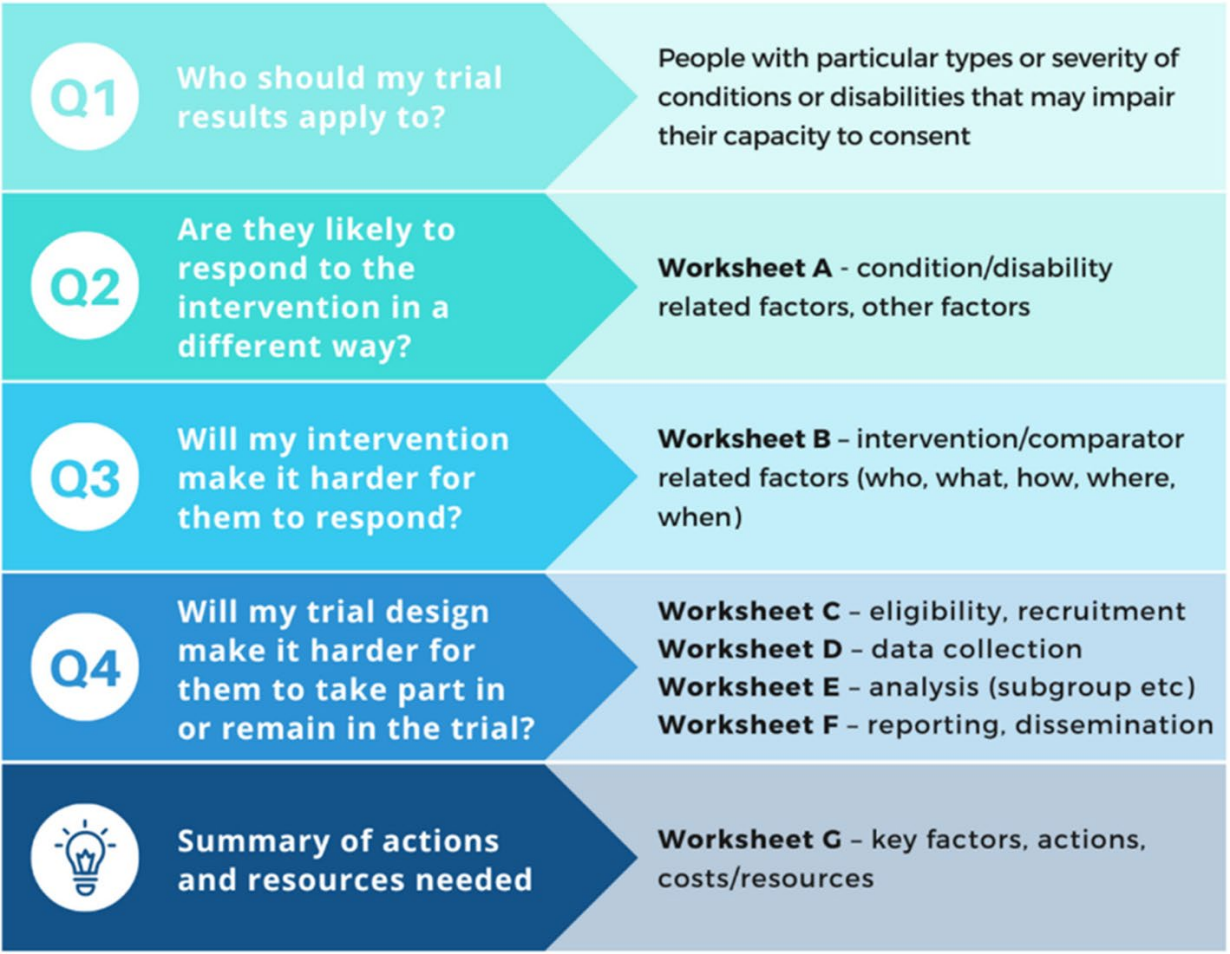
Structure of the INCLUDE Impaired Capacity to Consent Framework

The INCLUDE Impaired Capacity to Consent Framework is an editable document (Microsoft Word) containing two parts (Fig. 3). A set of four key questions help researchers identify which groups of people with impairing conditions should be included in their trial, and whether particular aspects of their condition, the intervention being tested, or the way the trial has been designed will affect their ability to take part. For each question, there are worksheets to help researchers answer the questions and identify what actions and resources are needed, with signposting to information and resources on capacity and consent [38] including summaries of the ethical and legal frameworks and practical guidance such as how to assess capacity and create accessible information sheets.

The worksheets cover areas including eligibility criteria, accessibility of trial information, informed consent arrangements, where and how data are collected, and how results are analysed and shared with these groups. Researchers then summarise the actions that could improve inclusivity in their trial, and any relevant resources needed, using the links to further information provided. Instructions for researchers about when the framework should be used, who by, and how much time and resource is involved are provided in Table 7 below.

**Table 7:** Instructions for using the INCLUDE Impaired Capacity to Consent Framework

**Who should use the framework**	
1. **Trial teams should use the framework as part of a collaborative process** — the framework is intended to be used by trial teams in partnership with patient and public partners (and other stakeholders) to ensure that the involvement of adults with a condition or disability that may impair their capacity to consent is considered at the trial design stage. 2. **The framework should be used by experienced and less experienced teams** — while the framework may cover issues that some trial teams had already considered, the worksheets will help to highlight issues consistently across trials and for all trial teams regardless of their experience through raising issues that teams have often not previously considered.	
**Which trials and populations should the framework be used with**	
3. **The framework should be used for all populations who experience impaired capacity** — impaired capacity may be due to the condition or disability that is the focus of the trial or may be co-existing with the condition or disability that the trial is focused on. The impairment may be long-term, a temporary or acute impairment where the intervention being tested cannot wait for the person to recover capacity, or the person’s capacity may fluctuate. While the worksheets ask trial teams to think about possible differences between groups who may experience impaired capacity, it is important to remember that no group is homogenous so there are also ‘within group’ differences, and there will be intersectionality between these and other factors or personal characteristics. 4. **Regardless of the focus of the trial** — the framework should be used when developing trials where the capacity-affecting condition/disability is the focus of the trial (e.g. dementia care), trials where the focus is another condition but cognitive impairment is highly prevalent in the population of interest (e.g. management of infections in care home residents), or where it may affect a small but important proportion of the overall population (e.g. diabetes prevention in high-risk populations which includes people with a learning disability). 5. **The framework may be useful for different types of research** — the framework is intended to be used for clinical trials, but it may also be useful for designing other types of studies and questions/sections that are not relevant can be left out. Some questions/sections will require interpretation to apply to the particular trial context, for example there are specific legal frameworks governing research involving adults who lack the capacity to consent which vary depending on the type of research (i.e if it is a clinical trial of a medicine or not, whether it is classed as emergency or nonemergency research) and where the trial is being conducted [39]. 6. **To identify any issues and the resources needed to address them** — throughout worksheets A–F, there are areas to note where any actions the trial teams may need to take in order to address the issues they identify. The final worksheet G provides a space to summarise these actions and any resources/costs needed to enable the participation of adults with impaired capacity to consent. For example, actions may include using tools such as the Consent Support Tool [40] to assess potential participants’ communication and support needs, creating accessible information about the trial, and ensuring research nurses or others provide tailored support to help meet individuals’ information and decision support needs and maximise their ability to contribute to decisions about participation. However, there are resource implications for purchasing the tool, time for developing accessible information, and ensuring research nurses have the time and skills to support people to participate in consent decisions. Ensuring adequate resources will require planning and justification at the funding application stage.	
**When the framework should be used**	
7. **The framework should be used at the earliest opportunity** — while it is intended to be used during early stages of trial design such as during funding applications, the framework can be completed iteratively. The questions posed in the framework can form the basis of discussions about the trial design, with the framework document being used to record the outcome of the discussions and actions required. The completed framework can be updated or referred back to at any point. 8. **Once the legal arrangements have been reviewed** — researchers should review the legal arrangements that will apply to their trial prior to completing the framework by reviewing the appendix which provides more information on the legal definition of capacity and the legal arrangements for including adults with impaired capacity to consent in research. 9. **Revisited during the design and conduct of a study** — it can be revisited during further trial development and will be particularly helpful when drafting the protocol, designing site training, and developing the application for ethical approval which is often seen as a challenge in studies involving adults who lack capacity to consent [17]. For example, question 1 which explores who should be included in the trial can help to justify why the trial should include adults lacking capacity and cannot be solely conducted with adults who are able to provide their own consent. 10. **Prior to ethics review** — exploring the processes for assessing capacity (including ensuring the personnel involved are appropriately skilled and experienced to do so) and identifying and approaching consultees and legal representatives (covered in worksheet C) will reassure ethics committees that the trial has been appropriately designed to include these populations.	
**Time and resources involved**	
11. **Time should be set aside to address inclusivity** — it may take a few hours to complete but this can be done over several occasions and not all sections may be relevant. Although it may increase time and work at the initial trial design phase, the framework supports researchers to fully consider issues and collaborate on solutions, which will enhance the quality of the funding application and can facilitate later stages such as when seeking ethical approval. 12. **Any associated costs can be included and justified in the funding application** — it may also increase the overall costs being requested [17] but will also help to justify how the inclusion of these otherwise ‘missing costs’ will ensure that the inclusive design is appropriately resourced. Using the framework will help ensure that inclusion is appropriately resourced, and funders are supportive of the use of the INCLUDE frameworks.	

Based on our observations of research teams implementing the framework in a diverse range of trial contexts during the facilitated workshops, feedback from stakeholders during and following the webinar, and subsequent discussions with other stakeholders, we developed a series of recommendations for research teams which provides a breakdown about what to consider when using the framework (Table 8).

**Table 8:** Recommendations for research teams using the INCLUDE Impaired Capacity to Consent Framework

Key questions	Worksheets	Topic or area of interest	Recommendations for research teams
Introduction	-	General background	• Review the legal frameworks governing research involving adults lacking capacity in the UK* *before* considering the framework questions as the justification for including adults lacking capacity will need to be clearly articulated when seeking approvals etc. Consider whether the study is connected with an impairing condition affecting participants who are unable to consent, or with the treatment of the condition. Could it be carried out as effectively (i.e. meet the research objectives) if it was confined to research participants able to give? • If the trial will involve different jurisdictions, familiarise yourself with the relevant legal frameworks — particularly if it is emergency research as the arrangements will vary considerably. Bear in mind that dual ethical approvals may be required • Public involvement (including families and carers where appropriate) is essential throughout
Q1. Who should my trial results apply to?	-	People with particular types or severity of conditions or disabilities that may impair their capacity to consent	• The framework will not help you to answer Q1 — you will need to explore the evidence about who makes up the target population. Consider who would be likely to receive the intervention if it is implemented into routine care. For example, who could benefit from the intervention if effective, or not having it if found to be ineffective? Are they likely to have conditions or disabilities that may impair their capacity to consent? What types or severity of conditions/disabilities are particularly relevant to consider? • Bear in mind that the capacity-affecting condition/disability may be long-term (e.g. learning disabilities), progressive in nature (e.g. dementia), be associated with an acute event (e.g. stroke, cardiac arrest) – or it may be a combination of these (e.g. a hip fracture in someone living with dementia) in which case multiple perspectives may need to be considered • Consider whether routine care pathways, clinician equipoise, or other factors may be different for these particular populations
Q2. Are they likely to respond to the intervention in a different way?	Worksheet A	Factors relating to the impairing condition/disability, plus other relevant factors such as ethnicity	• Q2 will be relevant to some trials more than others depending on the nature of the intervention/comparator • Consider whether participants’ ability to respond to or engage with an intervention will affect how effective the intervention will be. For example, people with cognitive impairment may find it difficult to engage with a self-management intervention • Consider whether the nature of impaired capacity (e.g. an acute event, progressive) may affect their ability to respond or engage. Might capacity to consent change over time (e.g. loss of capacity, regaining capacity)? What impact might that have? • In addition to cognitive disabilities, consider whether other disabilities that affect communication (e.g. aphasia, hearing or visual impairments) may affect participants’ ability to respond to the intervention, and consider how the trial design should take account of this • Consider whether other relevant factors or characteristics may affect participants’ ability to respond to the intervention, e.g. any cultural or language-related factors
Q3. Will my intervention make it harder for them to respond?	Worksheet B	Factors relating to the intervention/comparator (*including what the intervention/comparator is and where, when and who will be delivering it*)	• Q3 will be relevant to some trials more than others depending on the nature of the intervention/comparator • Consider whether participants’ ability to respond to or engage with an intervention will affect how effective the intervention will be. For example, an exercise intervention that requires participants to attend a programme of activities at an unfamiliar venue may be difficult to access for people with cognitive impairment • Depending on the nature of the intervention, prior work to explore whether the intervention is feasible for people with cognitive impairment may be needed
Q4. Will the trial design make it harder for them to take part in, or remain in, the trial?	Worksheet C	Eligibility and recruitment (*including consent*)	• Q4 will be relevant to all trials — and to many other types of research studies. Working through worksheets C–F will provide a systematic approach to working through the design of your trial • Consider whether eligibility criteria should focus on (in)capacity to consent or participants’ ability to engage with the intervention • If the trial will involve people who lack capacity to consent, familiarise yourself with the relevant legal frameworks* including assessing capacity, involving personal consultees and legal representatives (and nominated consultees and professional legal representatives where needed), and the additional documents that will be required • Consider how people with cognitive impairment might be given the opportunity to participate. For example, if an expression of interest is required via a website or return of a letter how might people be supported to do so? • Consider how participant information could be developed in ways that will enable people with cognitive impairment to receive and access it. Could this be in alternative formats? Who should be involved and how would this be resourced? • Consider whether participants’ ability to consent might change during the trial — what processes for re-assessing capacity and involving others might be needed? Consider which additional documents will be required. For example, someone enrolled without prior consent following a cardiac arrest may regain capacity to provide their own consent to continue in the trial. Someone living with dementia who provided consent at the outset may lose capacity during a trial and require the involvement of a consultee or legal representative
	Worksheet D	Data collection (*what, who, how and where will data be collected)*	• Worksheet D will depend on the nature of the data being collected. For example, whether any patient-reported outcome measures (PROMs) are being used and how they will be completed • Depending on the methods of data collection, prior work to explore whether it is feasible for people with cognitive impairment may be needed. For example, are proxy-completed versions required (and are there validated versions)? What happens about the continued use of data if a participant enrolled without prior consent dies before consent has been obtained? • Consider whether enabling a participant to nominate a study partner may be helpful in supporting participation and data collection
	Worksheet E	Analysis	• Consider how processes for reporting adverse events will include people who may be unable to self-report • Consider whether there may be any variation between groups who are able to consent and those who may not be able to consent. If there is a combination of self-reported and proxy-reported data how will these be analysed? Should there be planned subgroup or interim analyses, or any stopping triggers?
	Worksheet F	Reporting and dissemination	• Consider how and where trial results can be shared in ways that will enable people with cognitive impairment to receive and access them. Could this be in alternative formats? Who should be involved and how would this be resourced?
Summary of actions and resources needed	Worksheet G	Key factors identified and the actions and costs/resources needed	• This is your opportunity to recap on issues identified through the key questions and worksheets, outline the proposed actions or considerations to address them, and consider what costs or resources are required (if any) • Complete this worksheet as you go through the framework to form a summary of the discussions and decisions • Use this summary to justify the costs being requested in a funding application

*See Appendix 1 of the INCLUDE Impaired Capacity to Consent Framework for a summary of the legal frameworks governing research involving adults lacking capacity in the UK

## Conclusions

Addressing consent-based recruitment bias will ensure that people with impaired capacity to consent have an equitable opportunity to participate in research, and for the interventions they receive to be evaluated as safe and effective for them. Inclusion in research is essential in order to improve care for this under-served population and to reduce the health inequalities they experience. The INCLUDE Impaired Capacity to Consent Framework is intended to help researchers to design and conduct trials that are better quality and more inclusive of this population. Further work is needed to evaluate the effectiveness of the framework and identify implementation factors, to understand the multi-level contextual factors affecting its implementation in specific trial contexts [41], and to explore the wider intersectional factors affecting this and other under-served groups. The development and implementation process may serve as a guide to groups developing similar frameworks or tools that are intended to support researchers to design more inclusive research.

### Supplementary Information

**Additional file 1.** INCLUDE Framework User Guide.

**Additional file 2.** INCLUDE Framework FAQs.

## Data Availability

The NIHR INCLUDE Impaired Capacity to Consent Framework and accompanying resources are available online at: https://www.capacityconsentresearch.com/include-impaired-capacity-to-consent-framework.html

## Abbreviations

BSG: British Society of Gerontology
CRN: Clinical Research Networks
EOI: Expression of interest
HCRW: Health and Care Research Wales
IS: Implementation science
TCWG: MRC-NIHR Trials Methodology Research Partnership’s Trial Conduct Working Group
TMRP: MRC-NIHR Trials Methodology Research Partnership
MRC: Medical Research Council
NIHR: National Institute for Health and Care Research
NPT: Normalisation Process Theory
RCT: Randomised controlled trial
REC: Research ethics committee
UKTMN: UK Trial Management Network
