# Supplementary material for: Improving the inclusion of an under-served group in trials: development and implementation of the INCLUDE Impaired Capacity to Consent Framework
Source: Trials. 2024 Jan 25;25:83. doi: 10.1186/s13063-024-07944-x (PMC10809528; doi:10.1186/s13063-024-07944-x)
Supplement: Supplementary file 1 — Additional file 1. INCLUDE Framework User Guide. [file 13063_2024_7944_MOESM1_ESM.pdf]

Using the **INCLUDE** Impaired Capacity to Consent Framework

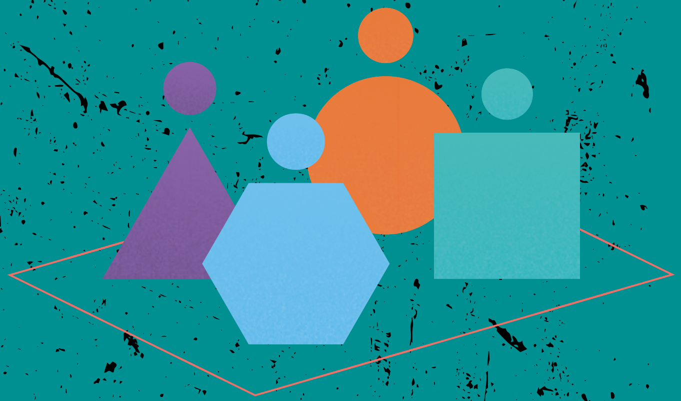

1
Who should trial results apply to?

Trial teams need to do everything possible to make their trial relevant to the people for whom the results are intended to apply, particularly for groups who are under-served by research (see **NIHR INCLUDE project**).

Tools are available to help researchers to design more inclusive trials, such as the INCLUDE Ethnicity Framework in relation to involving ethnic groups. This **INCLUDE Impaired Capacity to Consent Framework** aims to help trial teams think specifically about whether adults with impaired capacity to consent should be included in their trial for its results to be widely applicable, and what challenges there may be to making this possible. Having identified potential challenges, the team can then consider ways to reduce those challenges. For this to work, the Framework should be used at the trial design stage before funding is in place if possible and revisited when later developing the trial.

2
Why include people with impaired capacity to consent?

People who lack the capacity to consent for themselves have been recognised as an **under-served group**. The importance of including under-served groups in research is increasingly being highlighted by research funders. For example, **NIHR HTA Stage 2 guidance** requires applicants to demonstrate how inclusivity has been considered and addressed in their proposal, including the steps taken to ensure the research sample is representative of the population the study is targeted at.

Applicants are required to explain who they are planning to recruit to ensure inclusivity of study participants and justify and explain any exclusions. Excluding people with cognitive impairment from trials may mean the results are not applicable to them and it denies these groups the opportunity to participate in and benefit from research.

The framework is intended to apply to **all populations with conditions or disabilities** which may impair their capacity to consent to the trial (or who may lose capacity during the trial), however each trial and population will differ. Capacity may be impaired as a result of the condition or disability that is the focus of the trial, or the impairing condition or disability may be co-existing with the condition that the trial is focused on.

It may arise from an acute event leading to a sudden loss of capacity, from a long-term condition or disability, or be an acute event that happens to someone with a long-term condition or disability. In some circumstances, capacity can fluctuate or be lost or gained during the trial.

There are specific **legal frameworks** governing research involving adults who lack capacity to consent. These vary depending on the type of research (i.e if it is a clinical trial of a medicine or not, whether it is classed as emergency or non-emergency research) and where the trial is being conducted. See **Appendix 1** for more information on the legal definition of capacity and the legal arrangements for including adults with impaired capacity to consent in research. You may want to review the legal arrangements that will apply to your trial before completing the framework.

To download the INCLUDE Impaired Capacity to Consent Framework and access Appendix 1: **www.capacityconsentresearch.com**

CARDIFF UNIVERSITY PRIFYSGOL CAERDYDD

Trials Methodology TMRP Research Partnership

TRIAL FORGE

SUPPORTED BY NIHR | National Institute for Health and Care Research

3 What are the Key Questions and Worksheets?

The INCLUDE Impaired Capacity to Consent Framework has two elements:

1. Four Key Questions
2. Worksheets to help trial teams think through the answers to the Key Questions

The **Key Questions** are generic across all populations who may be under-served by research while the worksheets focus specifically on populations with impaired capacity to consent *. Once Key Question 1 (What groups should my results apply to?) has been answered - and this will require thinking through prior to using the framework - trial teams will know who needs to be in their trial. The remaining three questions ask trial teams to think through potential challenges to involving the people identified by Question 1 (see diagram below). These potential challenges include factors related to the intervention and aspects of trial design and delivery such as opportunities to participate, consent procedures, delivery of the intervention, and methods of data collection and analysis.

Diagram of INCLUDE Impaired Capacity to Consent Framework

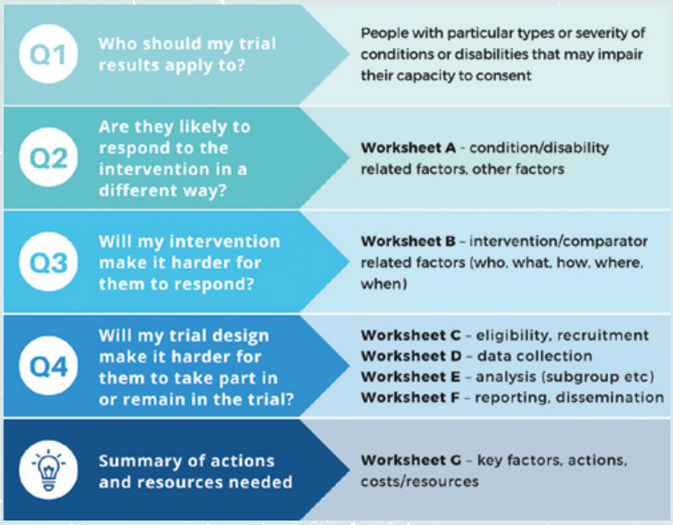

Answering these questions can be tricky, and every trial will have its own challenges. To help with this, we have developed the **worksheets** which give pointers to the sort of things to think about when answering Key Questions 2 to 4, any actions or considerations that might be needed, and to provide links to further resources. There is a hyperlink from Questions 2-4 to the corresponding worksheets.

4 Who should complete the Key Questions and Worksheets?

The framework is intended for use in trials but may be useful to consider for other types of studies. Using the framework is a collaborative *team effort*, not the task of an individual, and it is important to **include public involvement contributors**. It may take a few hours to complete but this can be done over several occasions and not all sections may be relevant. The completed framework can be updated or referred back to at any point e.g when developing the protocol, ethics application, and site training.

Although applying the framework will take additional time, it will enhance the quality of the funding application and facilitate these later development activities.

Using the framework will help ensure that inclusion is appropriately resourced. This may increase the overall costs being requested but it will help justify these otherwise 'missing costs' and funders are supportive of the use of INCLUDE frameworks.

5 Who has developed the Framework?

The Framework has been developed by members of the Inclusivity theme sub-group of the MRC-NIHR Trial Methodology Research Partnership's Trial Conduct Working Group. It is supported by the National Institute for Health Research (NIHR) INCLUDE initiative. See the Acknowledgements for a list of contributors.

* Other group-specific worksheets are available depending on the groups identified e.g see the **INCLUDE Ethnicity Framework**

For more information contact:
Dr Victoria Shepherd, Cardiff University **ShepherdVL1@cardiff.ac.uk**
