# Supplementary material for: Improving the inclusion of an under-served group in trials: development and implementation of the INCLUDE Impaired Capacity to Consent Framework
Source: Trials. 2024 Jan 25;25:83. doi: 10.1186/s13063-024-07944-x (PMC10809528; doi:10.1186/s13063-024-07944-x)
Supplement: Supplementary file 2 — Additional file 2. INCLUDE Framework FAQs. [file 13063_2024_7944_MOESM2_ESM.pdf]

NIHR INCLUDE Impaired Capacity to Consent Framework FAQs

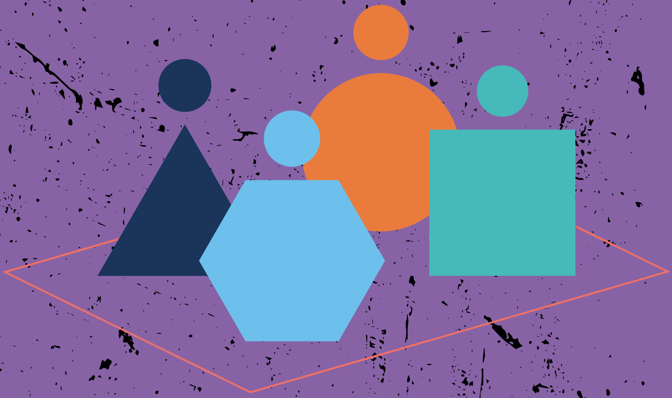

Q1 Does the framework explain the different legal provisions across the different legal jurisdictions of the UK?

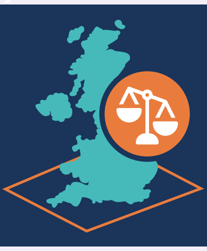

The framework is designed to be generic across populations and settings. The legal frameworks governing research involving adults who lack capacity to consent vary across the different UK jurisdictions and by the type of research (i.e whether it is classified as a clinical trial of an investigational medicinal product or not). The **introductory section** of the framework signposts researchers to **Appendix 1** which contains a summary of the requirements and terminology used in the different legal frameworks. This includes who is involved in decisions about adults who lack capacity to consent, how the decision should be made, and links to more information. Researchers are encouraged to review these prior to using the framework to design their study as these arrangements will need to be incorporated into the design.

Worksheet A also provides a reminder to consider whether the nature of participants' capacity and how it might change over their time in the study, and to review the different legal frameworks accordingly using the summary in Appendix 1.

The impact of the different legal frameworks that researchers need to consider in their trial are threaded throughout the framework. Questions about the consent processes and documents that might be required are contained in **Worksheet C**, and questions about whether participants will remain in the study if capacity is lost during the study is contained in **Worksheet E**.

Throughout the framework researchers are signposted to a **website of resources** (**www.capacityconsentresearch.com**) which includes pages about the legal frameworks and how the requirements apply in different studies with downloadable summaries.

Q2 Does the framework include guidance for emergency research conducted without prior consent?

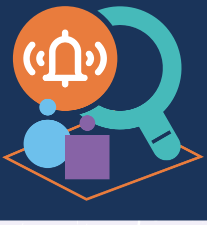

The framework is designed to be generic across populations and settings, including emergency and non-emergency research, although the issues that need to be considered will vary according to the context. The **introductory section** of the framework signposts researchers to **Appendix 1** which contains a summary of the requirements used in the different legal frameworks, including research conducted in emergency situations which varies in different jurisdictions. Researchers are encouraged to review these prior to using the framework to design their study as these arrangements will need to be incorporated into the design.

The impact of the different legal frameworks that researchers need to consider in their trial are threaded throughout the framework. For example, questions about the consent processes and documents that might be required, including for emergency research conducted without prior consent, are contained in **Worksheet C**.

Worksheet E covers aspect of retention, including the ability for participants to remain in the trial if capacity is lost, whether data will be used if the team are unable to obtain retrospective/deferred consent or in event of death or withdrawal, and highlights that whether consent is considered to survive any loss of capacity depends on the relevant legal framework.

Throughout the framework researchers are signposted to a **website of resources** (**www.capacityconsentresearch.com**) which includes information about the legal frameworks and how the requirements apply to emergency research with links to resources including a patient video explaining 'deferred consent'.

Cardiff University / Prifysgol Caerdydd
Trials Methodology Research Partnership (TMRP)
TRIAL FORGE
SUPPORTED BY NIHR | National Institute for Health and Care Research

Q3 Does the framework apply differently to different trial phases, or different types of research studies?

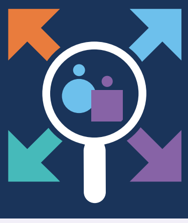

The framework is intended to be used to design clinical trials. However, we anticipate it is also useful when designing non-interventional studies and other types of research. The questions which specifically relate to the intervention or comparator (e.g Q2 and Q 3) may not be directly applicable but is likely that **Q4** and **Worksheets C-F** which relate to the design aspects of the study will be widely applicable.

The actions needed to address issues raised in these different types or stages of research will vary. For example, participating in a qualitative interview may be difficult for some people with cognitive impairment, and alternative methods, tools and approaches may be needed. This could include using photographs or objects that are meaningful to the person to help elicitation, or participatory research. The **website of resources** (**www.capacityconsentresearch.com**) includes pages about inclusive research and data collection.

Q4 Should separate feasibility studies be carried out to understand if people who lack capacity to consent can not only be recruited, but are able to complete/adhere to the intervention?

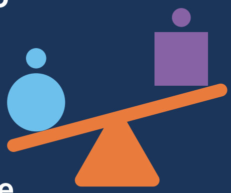

Inclusivity and diversity must be designed into studies from the outset. Feasibility studies play an important role in exploring whether aspects of the proposed trial design are feasible for the population concerned. This may include the intervention (if relevant and if it not already established in previous work) as well as recruitment and retention arrangements and data collection methods. Using the framework to design the feasibility study provides an opportunity to **identify and explore feasibility issues around the intervention/ comparator** as well as those around **recruitment and consent processes and data collection**. Aspects of the trial can then be amended prior to designing and conducting a larger scale trial.

Worksheet A encourages researchers to consider how any factors relating to participants' capacity-affecting condition or disability might influence their response to (or engagement with) the intervention/comparator.

Worksheet B enables researchers to consider how the intervention and/or comparator (including how they are provided), might make it harder for some groups in the population to respond to (or engage with) it. The factors listed in the worksheet are taken from the TIDieR guidelines for reporting interventions (**www.equator-network.org/reporting-guidelines/tidier**).

Worksheets C-F cover aspects of the trial design, including whether the data collection methods are appropriate and accessible for people with cognitive impairment who may encounter difficulties with self-reporting or whether alternative methods such as proxy reported outcomes are needed.

Q5 Do the worksheets provide guidance for working with relatives as personal consultees or personal legal representatives, or with staff members acting as nominated consultees or professional legal representatives?

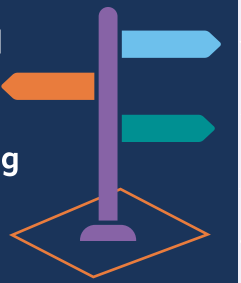

The framework does not explicitly include guidance for working with consultees and legal representatives, but the questions raised in the framework enables researchers to consider who may need to be involved, how they might be identified and contacted, and what information documents should be developed for them.

The **introductory section** of the framework signposts researchers to **Appendix 1** which contains a summary of the arrangements for identifying and involving consultees and legal representatives which varies in different jurisdictions and links for more information about how to work with them (e.g MCA Code of Practice **https://bit.ly/3XU8MRn** and Guidance on Nominating a Consultee **https://bit.ly/3R7IdWE**). Researchers are encouraged to review these prior to using the framework to design their study as these arrangements will need to be incorporated into the design.

Throughout the framework researchers are signposted to a **website of resources** (**www.capacityconsentresearch.com**) which includes information about the legal frameworks and how consultees and legal representatives are involved. The website also contains a **publications page** with studies reporting the experiences of relatives acting as consultees/legal representatives and an analysis of information sheets provided to personal and professional consultees/legal representatives. This may help researchers to understand consultees/legal representatives' informational and decisional needs.

Q6 Does the framework provide any guidance on retention in trials when including those with impaired capacity to consent?

Capacity can fluctuate over for time, and some participants capacity status may change during their time in a trial (i.e they may regain or lose capacity). This might be particularly anticipated in some populations or settings, and where a longer follow up period is planned. Prospectively planning for these situations will **avoid amendments** to the trial design (and so seek approvals for these amendments) at a later point and **reduce loss to follow up**.

The summary of the legal frameworks in **Appendix 1** includes the different arrangements for loss of capacity during research.

Worksheet E encourages researchers to consider whether participants are able to remain in the trial if capacity is lost, and about the use of data if the participant dies (including if the research team are unable to obtain consent for participants enrolled without prior consent or where a professional has acted as a consultee or legal representative). A range of information sheets to reflect these changes in consent arrangements will be needed (e.g a 'regained capacity' information sheet to reflect that the participant is agreeing to remain in the study rather than providing consent at the outset).

The framework signposts to the **website of resources** (**www.capacityconsentresearch.com**) for more information about the legal frameworks and how they govern the loss and regaining of capacity, including the page on 'research conduct'.

Q7 Do you have examples of completed frameworks used in other studies to look at?

Yes, the website has a **library of completed frameworks** that can be used as a reference (**www.capacityconsentresearch.com/include-impaired-capacity-to-consent-framework.html**). This includes trials in a range of settings, populations, and contexts, and involving different types of intervention. The intention is to expand this resource and we are keen to hear from researchers about any particular types of trials/populations they would like to be included, or if they would like to contribute their completed frameworks to the library (they can be anonymised if preferred).

Q8 When and how should capacity to consent be assessed? Is consent required prior to conducting a capacity assessment?

Mental capacity legislation has a number of underpinning principles. There is a legal presumption that a person has capacity to make a decision, unless there are concerns otherwise. Capacity is time and decision-specific, and so a person may have capacity to make some decisions and not others. If there are concerns that someone lacks capacity to consent to a study, there may be steps that can be taken to support the person to make their own decisions e.g to delay the decision until they regain capacity or to provide supported decision-making. Capacity may need to be reassessed at various timepoints during a trial if there is an indication that capacity has altered. For example, a participant may regain capacity once an emergency is over and be able to provide their own consent to continue in the trial. Or a participant who is living with dementia and who provides consent at the outset may lose capacity during the trial and a consultee or legal representative may be required to advise about their continued participation. Re-assessment may be required whenever consent is ordinarily revisited during a trial, e.g prior to a procedure or activity, or at contact points with participants.

The **website of resources** (**www.capacityconsentresearch.com**) includes information about the process of assessing capacity and about supported decision-making. This includes resources on conducting remote capacity assessments which may be required for some trials that don't involve face to face contact.

Questions about consent and capacity assessment processes are contained in **Worksheet C**. If indicated, a capacity assessment should be carried out by the person who requires the decision to be made (e.g the member of the research team who is seeking consent) and documented. Those involved in caring for the person may be able to advise the researcher about the person's communication and capacity needs. The website has links to a range of tools available to help support communication with participants and maximise their ability to make or participate in a decision about participation, such as Talking Mats or the Consent Support Tool.

'Consent' is not required before an assessment of the person's capacity is carried out, where it forms part of the process for recruiting participants. If the person is receiving care in a setting such as a care home for example, the care staff may introduce the researcher to them.

Q9 Is there commitment from funders to provide additional funding to enable researchers to include all populations who might benefit from the research as inclusive trials may be more costly?

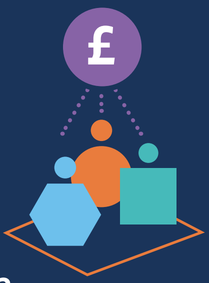

Some of the actions identified when designing a trial to be inclusive of adults with impaired capacity may require additional resources or costs. An example of where an action is needed is that additional documents may need to be drafted (e.g for consultees). An example of resource implications is that additional research nurse time may be needed to approach potential participants, assess capacity, and support participants to provide consent or approach a consultee or legal representative. Rather than viewing these as additional resources that are needed to recruit under-served population, they can be viewed as costs that are currently missing from funding applications.

The use of this (and other) frameworks when developing a funding application can help to **build in costs** associated with inclusivity from the outset, and to **provide justification** to the funders about what these costs are and why they have been included.

Many funders are committed to increasing inclusivity and diversity in research as a strategic priority. A number of funders **signpost applicants to the INCLUDE frameworks** (some are listed on the Trial Forge website (**www.trialforge.org/trial-forge-centre/impaired-capacity**).

Prospectively considering the barriers to recruitment and retention of participants (including those who lack capacity or whose capacity status may change during a trial) at the earliest design stage may **help reduce research waste** and so be associated with economic benefits.

Q10 It seems like the framework would need to be used at the outset, and then reviewed often and updated when appropriate. What advice is there for busy trial teams who already have so much going on?

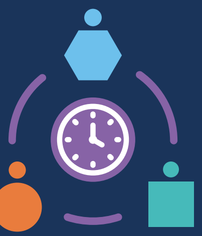

The framework does take time to work through and should be done collaboratively – including with patient and public contributors – at the earliest stage of developing a trial. Issues around inclusion of groups such as those with impaired capacity to consent can be complex, however considering any barriers and how to address them is likely to **improve the quality of the funding application**, increase the usefulness of the trial results, and save time later in the trial development process as many of the issues will have been **considered prospectively**. Responses to the questions can then be revisited when developing the protocol and other documentation for the trial. The summary of actions and resources identified in **Worksheet G** can be added to the 'justification of costs' section of the funding application.

Responses to **Q1** about why the trial should include people with impaired capacity can be used in the IRAS form section about why the trial cannot only be conducted with people who can provide their own consent when applying for **ethical approval**. This may improve the quality of the ethics application as many of the issues commonly raised during ethics review will have already been considered. It may also reduce delays in the ethics approval process arising from requests for additional information and documents.

The consent documents and processes outlined in **Worksheet C** (including who is involved, what documents are required, and what happens if capacity changes over time) can also be used in the ethics application and when developing participant documents for the trial.

A set of PowerPoint slides have also been developed which can be used to facilitate a **workshop discussion** by the research team as an alternative to completing the full framework document, or to support its completion, with the actions and resources identified then summarised in **Worksheet G**.

To download the INCLUDE Impaired Capacity to Consent Framework and access other resources: **www.capacityconsentresearch.com**

For more information contact:
Dr Victoria Shepherd, Cardiff University
ShepherdVL1@cardiff.ac.uk
